# The Mixture of Ferulic Acid and P-Coumaric Acid Suppresses Colorectal Cancer through lncRNA 495810/PKM2 Mediated Aerobic Glycolysis

**DOI:** 10.3390/ijms232012106

**Published:** 2022-10-11

**Authors:** Kaili Cui, Haili Wu, Jiangmin Fan, Lichao Zhang, Hanqing Li, Huiqin Guo, Ruipeng Yang, Zhuoyu Li

**Affiliations:** 1 The Key Laboratory of Chemical Biology and Molecular Engineering of Ministry of Education, Institute of Biotechnology, Shanxi University, Taiyuan 030006, China; 2 College of Life Science, Shanxi University, Taiyuan 030006, China; 3 Institutes of Biomedical Sciences, Shanxi University, Taiyuan 030006, China

**Keywords:** polyphenol, lncRNA 495810, PKM2, aerobic glycolysis, colorectal cancer

## Abstract

Polyphenol-rich foods are gaining popularity due to their potential beneficial effects in the prevention and treatment of cancer. Foxtail millet is one of the important functional foods, riches in a variety of biologically active substance. Our previous study showed that ferulic acid (FA) and p-coumaric acid (p-CA) are the main anticancer components of foxtail millet bran, and the two have a significant synergistic effect. In the present study, the clinical application potential of FA and p-CA (FA + p-CA) were evaluated in vivo and in vitro. The FA and p-CA target gene enrichment analysis discovered that FA + p-CA were associated with aerobic glycolysis. It was further shown that FA + p-CA remodel aerobic glycolysis by inhibiting the glycolysis-associated lncRNA 495810 and the glycolytic rate-limiting enzyme M2 type pyruvate kinase (PKM2). Moreover, PKM2 expression was positively correlated with lncRNA 495810. More interestingly, the exogenous expression of lncRNA 495810 eliminated the inhibitory effects of FA + p-CA on aerobic glycolysis. Collectively, FA + p-CA obstruct the aerobic glycolysis of colorectal cancer cells via the lncRNA 495810/PKM2 axis, which provides a nutrition intervention and treatment candidate for colorectal cancer.

## 1. Introduction

Colorectal cancer (CRC) is one of the most common malignant tumors of the digestive system, with high morbidity and mortality [1,2]. In recent years, with the progress of diagnosis and treatment technology, there have been many treatments applied in clinical practices [3]. However, the current treatment status of colorectal cancer is not promising. Therefore, it is not only necessary but urgent to find new treatments for colorectal cancer.

Colorectal cancer cells exhibit altered energy metabolism, particularly abnormal activation of glycolysis pathways [4,5]. Compared with normal cells, even in the presence of sufficient oxygen, CRC cells prefer to generate ATP through glycolysis, rather than completing oxidation through the tricarboxylic acid cycle [6]. This phenomenon is defined as the Warburg effect, also known as aerobic glycolysis. Enhanced aerobic glycolysis has been shown to promote the proliferation and metastasis of colorectal cancer cells [7]. Therefore, intervening of glycolysis could be an effective strategy for the treatment of CRC cancer.

Polyphenols, ubiquitous in plant-derived cereals, fruits, and vegetables, have been shown to prevent CRC by modulating glycolysis. Accumulating evidence suggests that resveratrol [8], chrysin [9], and kaempferol [10], dietary polyphenol from plant-derived cereals, fruits, and vegetables, inhibits glycolysis via reducing glycolysis rate-limiting enzymes, thereby inhibiting the proliferation of cancer. These studies suggest that polyphenols have the potential to be developed as excellent agents for remodeling glycolysis in CRC.

In recent years, the active ingredients in functional foods have received increasing attention as a rich source of potential anticancer drugs [11,12]. Millet is native to China and is one of the important functional foods, rich in a variety of biologically active substance. Studies in China and abroad have shown that millet bran is rich in polyphenols, and it was discovered that polyphenols from millet bran have a significant anti-colon cancer effect [13,14,15]. In previous studies, we found that ferulic acid (FA) and p-coumaric acid (p-CA) are the most abundant polyphenols in millet bran and were proved to synergistically inhibit colorectal cancer cells growth [16,17]. In this study, we uncovered the mechanism of FA and p-CA (FA + p-CA) remodel aerobic glycolysis of colorectal cancer cells via the lncRNA 495810/PKM2 axis. This study provides an important basis for tumor nutrition intervention and treatment of FA + p-CA on colon cancer.

## 2. Results

### 2.1. FA + p-CA Inhibit the Growth of Colorectal Cancer in CRC Cells and AOM/DSS CRC Mice

**Figure 1:**
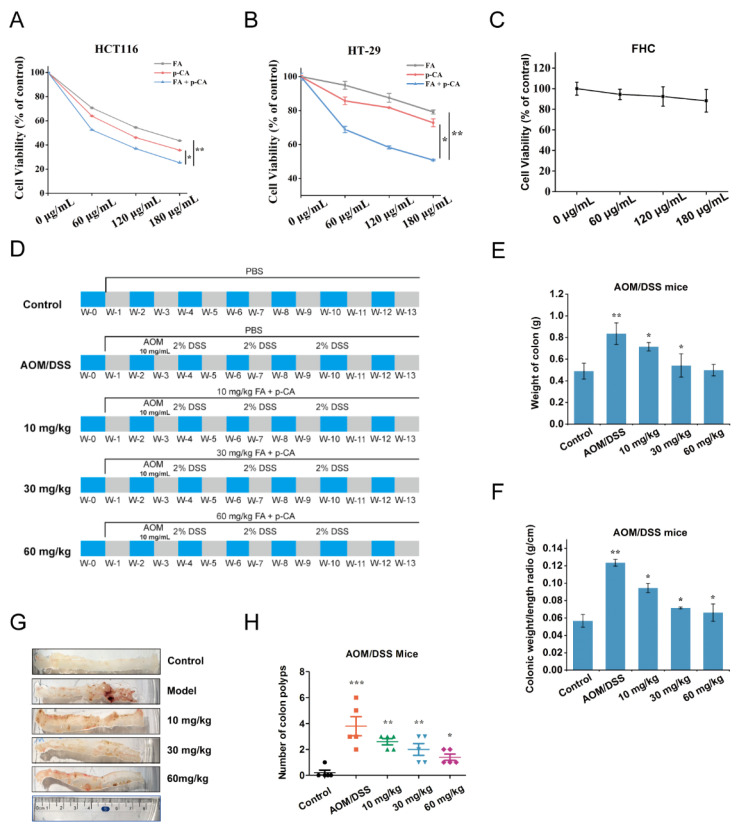
FA + p-CA inhibit the growth of colorectal cancer in CRC cells and AOM/DSS CRC mice. (**A**–**C**) The HCT116 (**A**), HT-29 (**B**) and FHC (**C**) cells were respectively exposed to increasing concentrations of FA, p-CA, and FA + p-CA for 48 h, the cell viability was then detected using MTT. The content ratio of p-CA and FA in the FA + p-CA was 1:1.13. (**D**) An overview of the experimental design of the AOM/DSS mice model. The mixture of FA + p-CA was intra-gastrically administered, and the content ratio of p-CA and FA was 1:1.13. (**E**) Weight of the colorectal in different groups in the 14th week was recorded. (**F**) Colonic weight/length ratio in different groups was detected. (**G**) Macroscopic structure findings of the colon in different groups. (**H**) Tumor numbers in the colon in different groups were annalistic. Data are represented as means ± SD (*n* ≥ 5). Compared to control, * *p* < 0.05, ** *p* < 0.01, *** *p* < 0.001.

To detect the inhibitory effects of FA + p-CA on CRC cells, p-CA and FA were artificially mixed at a ratio of 1:1.13 [16]. Following 48 h of treatment, the proliferation of HCT116 and HT-29 cells was dose dependently inhibited by 60, 120 and 180 μg/mL FA, p-CA and FA + p-CA (Figure 1A,B). Furthermore, the IC50 of FA + p-CA to HCT116 cells was 67.00 μg/mL, while the IC50 of FA and p-CA were 140 μg/mL and 103.186 μg/mL, respectively. These data indicated that the inhibition of the single component was significantly lower than the mixture, consistent with our previous results showing that FA + p-CA has a significant effect in inhibiting colon cancer cells [16]. According to this result, we selected FA + p-CA for subsequent studies. Then, we examined the cytotoxicity of FA + p-CA on human colon epithelial cells line (FHC) and no cytotoxicity was observed at concentrations below 180 μg/mL (Figure 1C). Subsequently, according to the process shown in Figure 1D, we constructed an AOM/DSS-induced CRC mice model and assessed the anti-CRC activity of FA + p-CA in vivo. The results specified that the CRC indices including weight of colon and colonic weight/length ratio in AOM/DSS mice were significantly increased compared to the controls. Moreover, following FA + p-CA treatment, the above indices of AOM/DSS mice decreased in a dose-dependent manner (Figure 1E,F). Furthermore, compared with the control, the number of tumor polyps in AOM/DSS increased significantly, while its in 10 mg/kg, 30 mg/kg and 60 mg/kg groups were significantly decreased in a concentration-dependent manner (Figure 1G,H). All these findings conclude that FA + p-CA exhibit inhibitory effect on colorectal cancer in vivo and in vitro.

### 2.2. FA + p-CA Involve in the Aerobic Glycolysis Pathway

**Figure 2:**
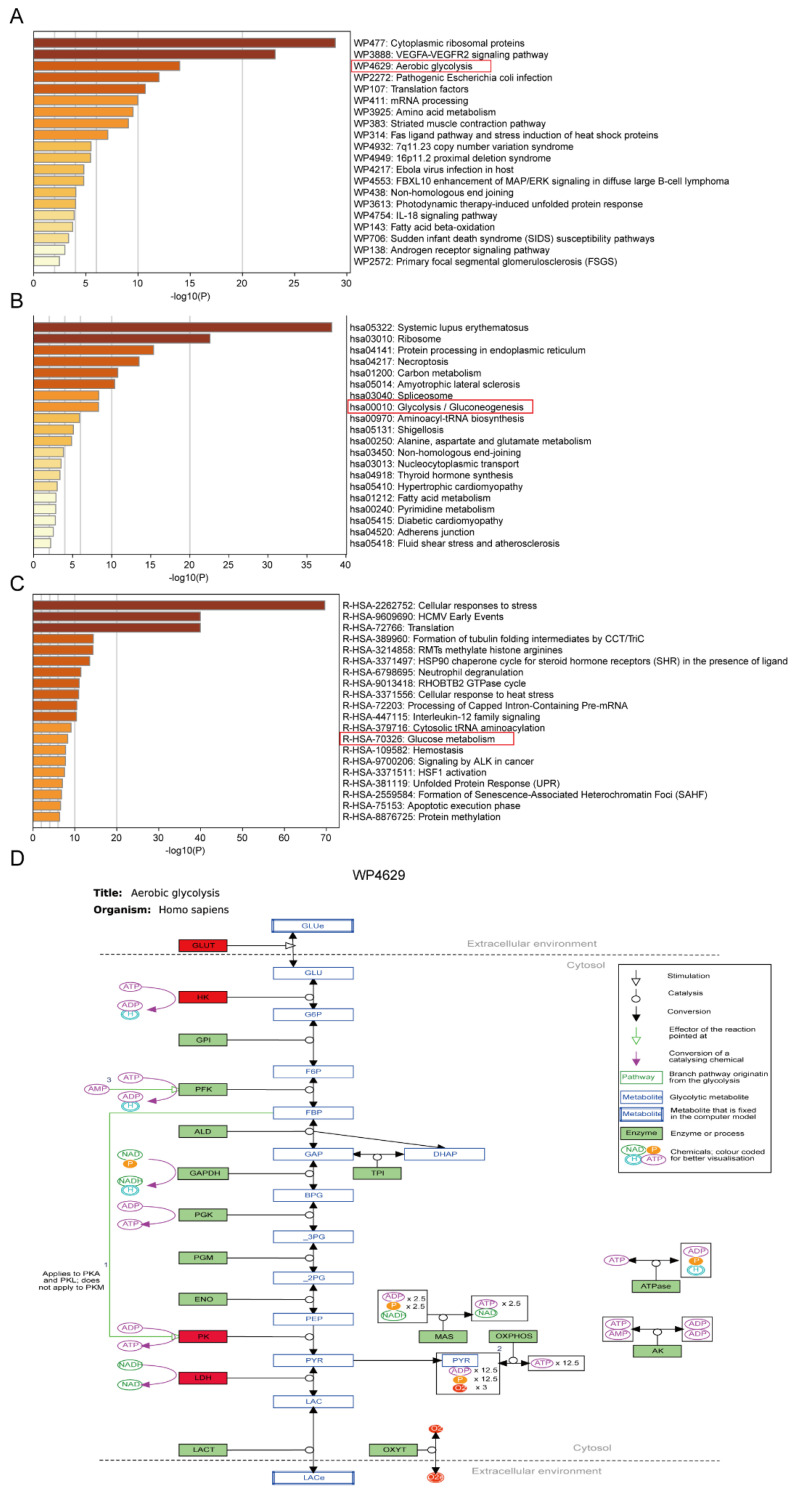
FA + p-CA are involved in the aerobic glycolysis pathway. The FA and p-CA target proteins were imported into the Metascape database for gene enrichment analysis. (**A**–**C**) Summary of enrichment analysis in WikiPathways (**A**), KEGG (**B**) and reactome pathway knowledgebase (**C**), colored by *p*-values, the color becomes lighter as the *p*-value increases. The red box shows that genes are enriched in the glycolytic pathway. (**D**) Aerobic glycolysis (WP4629) pathway involving FA and p-CA target proteins.

To explore the pathways regulated by FA + p-CA, the target proteins of FA and p-CA were identified by Solid-phase extraction assay combined with mass spectrometry. Then, the above target proteins were imported into the Metascape database (www.metascape.org) for gene enrichment analysis via WikiPathways. The results indicate that the FA + p-CA target genes were enriched in the aerobic glycolysis pathway (Figure 2A). They were enriched for glycolysis and gluconeogenesis pathways as analyzed by the KEGG (Figure 2B). Meanwhile, through the reactome pathway knowledgebase analysis, FA + p-CA target genes were enriched in the glucose metabolism pathway (Figure 2C). The above results indicate that FA + p-CA are associated with aerobic glycolysis.

### 2.3. FA + p-CA Obstruct the Expression of Glycolysis-Related lncRNA 495810

**Figure 3:**
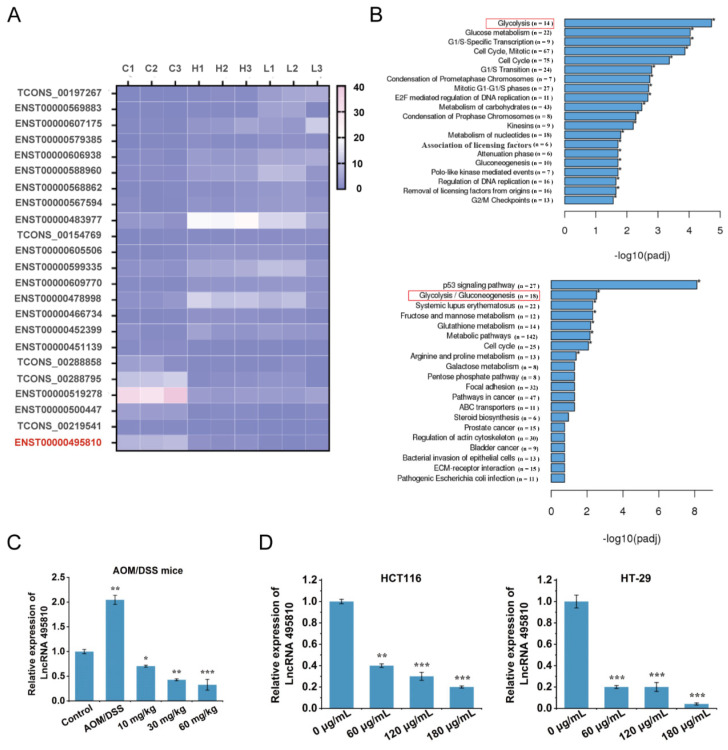
FA + p-CA inhibit the expression of glycolysis-related lncRNA 495810. (**A**) Cluster analysis showing differentially expressed lncRNA expression in the L groups and H groups compared with the C groups. The red letters show that lncRNA 495810 (named after its ENST ID: ENST00000495810) is the lncRNA of interest in this study. (**B**) KEGG and the Reactome pathway knowledgebase analysis between C groups and the L groups. The red box shows that genes are enriched in the glycolytic pathway. (**C**,**D**) The expression of lncRNA 495810 was measured in AOM/DSS mice (**C**), HCT116 and HT-29 cells (**D**) treated with FA + p-CA. Data are represented as means ± SD (*n* ≥ 5). * *p* < 0.05, ** *p* < 0.01, *** *p* < 0.001.

Recent studies have shown that lncRNAs act as small molecule target genes [18]. Small molecules alter the conformation of lncRNAs [19] or act as their inhibitors [20] to target and regulate their expression. To find out the involvement of lncRNA in the inhibitory effect of FA + p-CA, RNA-Seq was conducted to screen differentially expressed long non-coding RNAs in HCT116 cells treated with FA + p-CA. In total, 23 differentially expressed lncRNA were chosen with rigorous cutoff criteria: *p* < 0.05, |Log2 FoldChange| ≥ 2 (Supplementary Table S2). Among which, there were 16 lncRNAs up-regulated and 7 down-regulated lncRNAs in the L (60 μg/mL FA + p-CA) group and H (180 μg/mL FA + p-CA) group compared to C (0 μg/mL FA + p-CA) group (Figure 3A). Further, through kyoto encyclopedia of genes and genomes (KEGG) and reactome pathway knowledgebase analysis, the top enrichment pathway was the glycolysis pathway (Figure 3B). All these findings suggest that FA + p-CA is involved in aerobic glycolysis.

According to the result of RNA sequencing, lncRNA 495810 (named according to its ENST ID: ENST00000495810) attracted our attention due to it being the most downregulated one upon FA + p-CA treatment, which may be involved in glycolysis. To identify the consequence of RNA-Seq, the expression of lncRNA 495810 in AOM/DSS-induced CRC mice and different colon cancer cells was detected after exposure to FA + p-CA. The results indicated that the expression of lncRNA 495810 in the AOM/DSS group was increased, whereas additional treatment of FA + p-CA decreased its expression (Figure 3C). Corresponding to it, the lncRNA 495810 expression in CRC cells decreased along with the increased concentration of FA + p-CA (Figure 3D). Collectively, the data indicate that glycolysis-related lncRNA 495810 is suppressed by FA + p-CA.

### 2.4. FA + p-CA Weaken the Expressions of PKM2 to Block Aerobic Glycolysis

**Figure 4:**
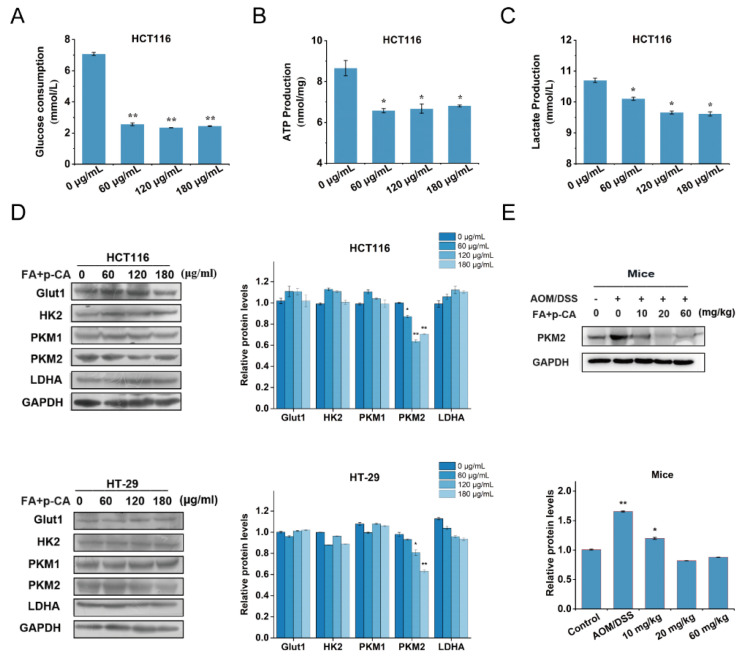
FA + p-CA weaken the expressions of PKM2 to block aerobic glycolysis. (**A**–**C**) Detection of glucose consumption (**A**), ATP generation (**B**) and lactate production (**C**) in HCT116 cells treated with different concentration of FA + p-CA (0, 60,120, 180 μg/mL) for 48 h. The data were presented as the mean ± SEM from three independent experiments. (**D**) HCT116 and HT-29 cells were treated with different concentration of FA + p-CA for 48 h, then the protein level of PKM2 was determined by western blot. Expression of GAPDH was used as internal control. (**E**) The expression of PKM2 protein was measured in AOM/DSS mice treated with FA + p-CA. Data represented as means ± SD (*n* ≥ 5). Compared to control, * *p* < 0.05, ** *p* < 0.01.

Further, to assess the regulatory effect of FA + p-CA on aerobic glycolysis in CRC, the glucose consumption, ATP generation and lactate production were detected after the HCT-116 cells were treated with different concentration of FA + p-CA. The results showed that all the above indices were decreased (Figure 4A–C). Additionally, the expression of a series of aerobic glycolysis related enzymes showed in Figure 2D (protein included in red squares) was measured in the HCT116 and HT-29. It was found that only PKM2, one of the rate-limiting enzymes of aerobic glycolysis, was notably suppressed by FA + p-CA (Figure 4D). Moreover, the effects of FA, p-CA, and FA + p-CA on PKM2 expression, which is its downstream effector, were examined. Consistent with its inhibitory effect on colorectal cancer, FA + p-CA inhibited PKM2 expression better than the single component (Figure S1A,B). In addition, the expression of PKM2 in AOM/DSS increased significantly, while its in 10 mg/kg, 30 mg/kg and 60 mg/kg groups were decreased (Figure 4E). Collectively, the results imply that FA + p-CA weakens aerobic glycolysis by restraining the expression of PKM2.

### 2.5. LncRNA 495810/PKM2 Axis Is Involved in the Inhibition Effect of FA + p-CA on the Aerobic Glycolysis

**Figure 5:**
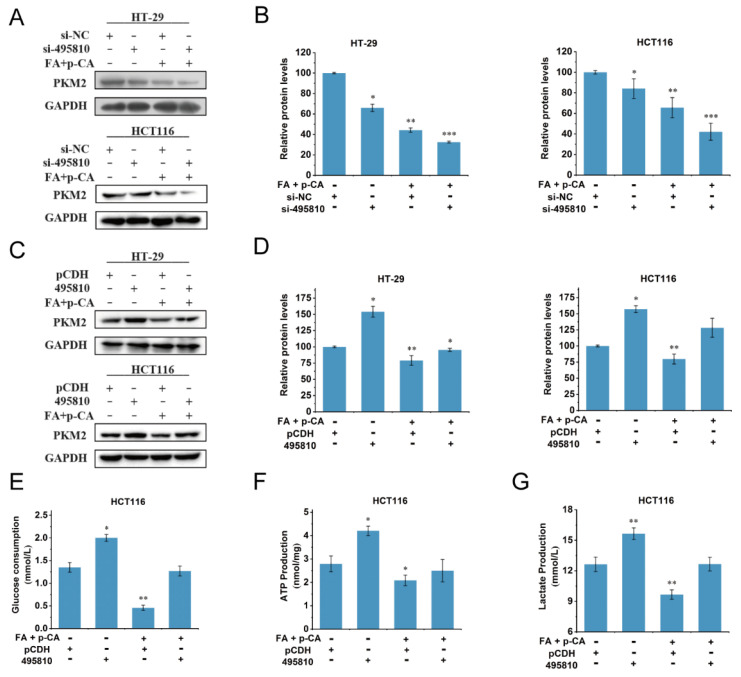
LncRNA 495810/PKM2 axis is involved in the inhibition effect of FA + pCA on the aerobic glycolysis. (**A**,**B**) At 24 h post-transfection with si-NC or si-495810, HT-29 and HCT116 cells were treated with 120 μg/mL FA + p-CA for another 48 h, the expression of PKM2 protein was checked by western blot (**A**). The densitometry analysis of relative protein expression (**B**). (**C**,**D**) At 24 h post-transfection with pCDH or 495810, HT-29 and HCT116 cells were treated with 120 μg/mL FA + p-CA for another 48 h. The expression of PKM2 protein was determined by western blot (**C**). The densitometry analysis of relative protein expression (**D**). (**E**–**G**) At 24 h post-transfection with pCDH or 495810, HT-29 and HCT116 cells were treated with 120 μg/mL FA + p-CA for another 48 h, glucose consumption (**E**), ATP generation (**F**) and lactate production (**G**) were discovered. Data are represented as means ± SD (*n* ≥ 5). * *p* < 0.05, ** *p* < 0.01, *** *p* < 0.001.

We, therefore, considered whether lncRNA 495810 could regulate PKM2. The results showed that silencing lncRNA 495810 expression declined, whereas ectopic lncRNA 495810 expression enhanced the expression of PKM2. The PKM2 expression was positively correlated with lncRNA 495810. We therefore hypothesized that FA + p-CA inhibit aerobic glycolysis via the lncRNA 495810/PKM2 axis. To test our hypothesis, the HT-29 and HCT116 cells were transfected with 495810 and treated with FA + p-CA. The results showed that lncRNA 495810 knockdown enhanced and overexpression abrogated the inhibitory effect of FA + p-CA on PKM2 (Figure 5A–D). Phenotypically, the inhibitory effect of FA + p-CA on glucose consumption, ATP production and lactate production were decreased when lncRNA 495810 was overexpressed (Figure 5E–G). The above data indicated that the lncRNA 495810/PKM2 axis is required for FA + p-CA inhibiting aerobic glycolysis.

## 3. Discussion

Recently, extensive attention has been paid to the search for efficient and non-toxic anti-CRC drugs or functional molecules from natural products [21,22,23]. Numerous epidemiological studies have shown that dietary patterns high in whole grains are associated with a lower risk of CRC [24,25]. Therefore, whole grain extracts may provide a safe and effective way to prevent the development of colon tumors in humans. Millet is nutritionally superior to rice and wheat because it contains high amounts of bioactive substances. In our previous study, it was demonstrated that FA and p-CA are the most abundant polyphenols in millet bran, and they exhibited synergistically anti-colon cancer activity in CRC cell models. However, CRC cell models cannot fully reflect the characteristics and effectiveness of antitumor drugs. In this study, the AOM/DSS CRC model, which is widely used in anti-colorectal cancer drug research [26], was used to further evaluate the anti-CRC effect of FA + p-CA. The results showed that FA + p-CA restrained the growth of tumors in AOM/DSS CRC mice (Figure 1).

Metabolic remodeling is one of the malignant features of tumor cells [27]. Therefore, intervening of glycolysis could be an effective strategy for the treatment of CRC cancer. In this study, the target proteins and regulated lncRNAs of FA and p-CA were obtained by Solid-phase extraction assay combined with mass spectrometry and RNA sequencing technology. Further, gene enrichment analysis found that it was enriched in the aerobic glycolysis pathway. The above results demonstrate the correlation of FA + p-CA with glycolysis (Figure 2 and Figure 3). Recently, plant polyphenols, such as kaempferol [10], resveratrol [28], quercetin [29], and hesperidin [30], have been reported to exert antitumor effects by reversing the Warburg effect. This study found that FA + p-CA treatment resulted in reduced glucose consumption, lactic acid accumulation, and ATP production, which showed that FA + p-CA exerts an inhibitory effect on glycolysis. Further, we also investigated the expression of key metabolic enzymes in glycolysis. Interestingly, we found that PKM2, the rate-limiting enzyme of glycolysis, was significantly inhibited by FA + p-CA (Figure 4). Above all, the data showed that FA + p-CA impair aerobic glycolysis by inhibiting PKM2.

Abnormal expression and function of lncRNA plays a key role in regulating tumor glucose metabolism remodeling [31,32,33]. It is reported that the expression of lncRNA PVT1 is up-regulated in tumors, which regulates HK2 expression by competitively binding to endogenous miR-143 in gallbladder cancer cells and promotes aerobic glycolysis of tumor cells [34]. In our studies, the data showed that a glycolysis-associated lncRNA 495810 was significantly restrained by FA + p-CA both in vivo and in vitro (Figure 3). Furthermore, the results showed that PKM2 expression was positively correlated with lncRNA 495810. More interestingly, the exogenous expression of lncRNA 495810 eliminated the inhibitory effects of FA + p-CA on glucose consumption, pyruvate accumulation, ATP production and PKM2 protein (Figure 5), suggesting that the lncRNA 495810/PKM2 axis mediated the blocking of FA + p-CA on glycolysis.

## 4. Materials and Methods

### 4.1. Chemicals

Ferulic acid (FA) and p-coumaric acid (p-CA) were purchased from Victory Biological Technology Co., Ltd. (Sichuan, China). The content ratio of p-CA and FA in the mix was 1:1.13 [16]. DMEM/F12 (HAM) 1:1 medium and fetal bovine serum (FBS) were purchased from Biological Industries (Kibbutz Beit Haemek, Israel); MTT and dimethyl sulfoxide (DMSO) were purchased from Solarbio (Beijing, China). The ATP assay kit was obtained from Beyotime Biotechnology (Shanghai, China). The lactate test kit was purchased from Nanjing Jiancheng Bioenigeering Institue (Nanjing, China). Antibodies for Glut1, HK2, LDHA, PKM2 and GAPDH were from ProteinTech (Chicago, IL, USA). Anti-PKM1 was purchased from Cell Signaling Technology (Danvers, MA, USA). The RNAiso Plus was purchased from Takara (Shiga, Japan). The TransScript First-Strand cDNA Synthesis SuperMix and TransStart Top Green qRT-PCR SuperMix were from TransGen (Beijing, China). The turbofect was from Thermo Scientific (Waltham, MA, USA). The glucose assay kit and HiPerFect Transfection Reagent were purchased from QIAGEN (Venlo, The Netherlands).

### 4.2. Mice Experiment

Male C57 BL/6 J mice (5 weeks old) were purchased from Beijing Weitahe Labor Animal Technology Co., Ltd. (Beijing, China). All mice were raised in SPF cages in the laboratory at the Animal Service Center of China Institute of Radiation Protection. Animal experiments were approved by the National Institutes of Health and the Animal Experiment Ethics Committee of Shanxi University (Shanxi, China) under the principles of laboratory animal protection. The mice were divided into five groups (*n* = 5 in each group): control group; AOM/DSS group; intervention group with different concentrations of FA + p-CA (10 mg/kg, 30 mg/kg, 60 mg/kg) (Figure 1D). The control group was given PBS. To construct a mouse model of CRC induced by azomethane (AOM)/dextran sulfate sodium (DSS), mice were injected with AOM (10 mg/kg, Sigma Chemical) for once, then two weeks later, followed by three cycles of 2% DSS (MP Biomedicals, Santa) treatment (1 week with DSS, 2 weeks without DSS). For the FA + p-CA intervention group, FA + p-CA (10 mg/kg, 30 mg/kg, 60 mg/kg) was intra-gastrically administered once every 2 days after acclimatizing a week, and the others were the same as the AOM/DSS group. At the end of the study, all mice were anesthetized with ether and tissues were immediately removed, weighed, and frozen in liquid nitrogen for further analysis.

### 4.3. Cell Line and Cell Culture

The colorectal cancer cell lines HT-29, HCT116 and SW620 were obtained from the Chinese Type Culture Collection (Shanghai, China). The cells were cultured in DMEM/F12 medium containing 10% FBS and placed in an incubator at 37 °C with 5% CO_2_.

### 4.4. Cell Viability Assay

Different colon cancer cells were seeded into 96-well plates, and after the cells were treated with different doses of FA + p-CA (60, 120 and 180 μg/mL) for 48 h, 10 μL MTT was added for 4 h, and the OD value at 570 nm was detected.

### 4.5. Metabolic Assays

The cells were inoculated into the 6-well plate, and the culture medium and cells were collected after treating with FA + p-CA. The medium was used to determine the contents of glucose and lactate, and the cells were used to determine the production of ATP. The methods of measuring glucose, ATP, and lactate followed the manufacturer′s instructions.

### 4.6. Western Blot Assay

The total protein was extracted by WBIP, and the protein concentration was measured by BCA assay. The equal amount of protein was separated by SDS-PAGE and transferred to PVDF membrane. The membrane was blocked for 40 min at room temperature with 5% skim milk and incubated with different primary antibodies overnight for 4 °C. Then, the membrane was incubated with rabbit/mouse second antibody at room temperature for 2 h. Finally, the change of protein expression was observed by exposure.

The mice intestinal tissue samples were taken from −80 °C, dissolved in a lysis buffer containing 1 mM PMSF (*w*/*v* = 1:7), and then ground with TissueLyser at 60 Hz for 2 min at 4 °C, then put on ice for 30 min. Centrifuge the lysate at 4 °C at 12,000 rpm for 15 min. The supernatant collected for protein concentration was determined with BCA detection kit. Next, the protein sample method is analyzed by Western blotting as described above.

### 4.7. LncRNA Sequencing

The LncRNA expression profiles of HCT116 cells upon different concentration of FA + p-CA (C group: 0 ug/mL, L group: 60 μg/mL FA + p-CA, H group: 180 μg/mL FA + p-CA) intervention were detected by RNA-seq. The LncRNA expression was accurately tested and corrected by Fisher and FDR values. The RNA-seq was conducted by Novogene Co., Ltd. (Beijing, China).

### 4.8. Quantitative RT-PCR Assay

The total RNA was extracted from cells with Trizol reagent, and 500 ng RNA was reverse-transcribed using EasyScript First-Strand cDNA Synthesis SuperMix. The LncRNA and mRNA levels were quantified using qRT-PCR. GAPDH mRNA was used as the control. The primers used are listed in Supplementary Table S1.

### 4.9. Vector Construction and siRNA

For the determination of siRNA transfection, the cells were inoculated into 6-well plates, and 10 nM siRNA was transfected with HiPerFect Transfection Reagent for 48 h according to the manufacturer′s scheme. For transient plasmid transfection, the plasmid was transfected into colon cancer cells using Turbofect reagent according to the manufacturer′s instructions. At 48 h after transfection, the cells were collected for subsequent analysis or determination. The siRNA sequences were as follows: lncRNA 495810-homo-112 (sense, 5′-CCAUACCAUCAAUGGUCAUTT-3′; antisense, 5′-AUGACCAUUGAUGGUAUGGTT-3′).

### 4.10. Statistical Analysis

The data were presented as the mean ± standard deviation of three independent experiments (Mean ± SD), data error bar indicated standard deviation. Single factor analysis of variance (ANOVA) was used for comparisons of means of ≥3 groups. Single variable comparisons were performed by Student′s *t*-test, the values of *p* < 0.05 indicated that there was a significant difference, while *p* < 0.01 were considered that the difference was highly significant compared with control.

## 5. Conclusions

**Figure 6:**
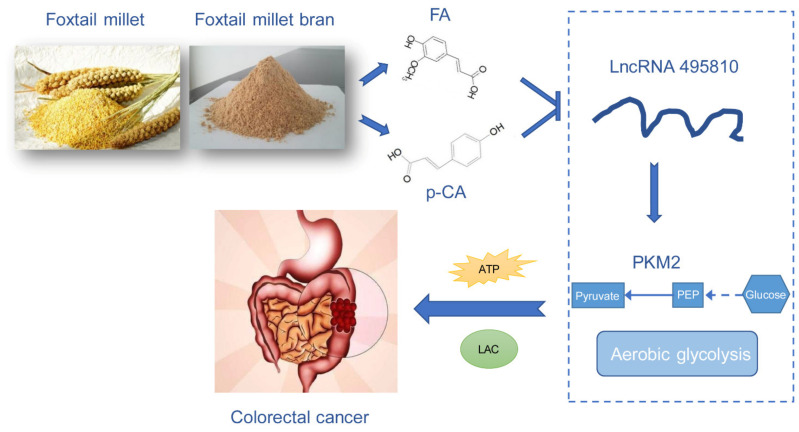
Schematic presentation indicates the metabolic mechanism by which FA + p-CA -induced anti-CRC effects. FA + p-CA reverse the aerobic glycolysis through the lncRNA 495810/PKM2 cascade, thereby inhibiting colon cancer.

In summary, we found that FA + p-CA exert the anti-tumor activity in CRC cells and AOM/DSS CRC mice. Moreover, FA + p-CA reverse the aerobic glycolysis through the lncRNA 495810/PKM2 cascade (Figure 6). Collectively, FA + p-CA have the potential as a new prophylactic and therapeutic candidate for colorectal cancer.

## Data Availability

All data needed to evaluate the conclusions in the paper are present in the paper.

## Supplementary Materials

The supporting information can be downloaded at: https://www.mdpi.com/article/10.3390/ijms232012106/s1.
