# Supplementary material for: The Mixture of Ferulic Acid and P-Coumaric Acid Suppresses Colorectal Cancer through lncRNA 495810/PKM2 Mediated Aerobic Glycolysis
Source: Int J Mol Sci. 2022 Oct 11;23(20):12106. doi: 10.3390/ijms232012106 (PMC9603647; doi:10.3390/ijms232012106)
Supplement: Supplementary file 1 [file ijms-23-12106-s001.zip › ijms-1966111-supplementary.pdf]

Supporting Information

Supplement Table S1. Primers Sequences Used for qRT-PCR

Genes	Sequences (5′−3′)	
**PKM2**	forward primer:	reverse primer:
	GCTGCCATCTACCACTTGC	CCAGACTTGGTGAGGACGATT
**lncRNA 495810**	forward primer:	reverse primer:
	CGTGGATTTGGGGATGGCTA	CTGGACCACTATCACCCAAGC
**GAPDH**	forward primer:	reverse primer:
	GCACCGTCAAGGCTGAGAAC	TGGTGAAGAACGCCAGTGGA

Supplement Table S2. Differentially expressed lncRNA in the L group and H group compared to group C.

	C&L		C&H	
**ID Number**	**log2FoldChange**	**p value**	**log2FoldChange**	**p value**
**TCONS_00197267**	3.28576	0.02125	5.87298	0.01905
**ENST00000569883**	2.08773	0.0215	4.47139	0.00065
**ENST00000607175**	2.99619	0.0099	3.83447	0.00315
**ENST00000579385**	2.84846	0.03315	3.08558	0.02925
**ENST00000606938**	1.91398	0.00045	2.80526	5.00E-05
**ENST00000588960**	1.98041	0.00015	2.70528	5.00E-05
**ENST00000568862**	1.84591	0.0022	2.62905	5.00E-05
**ENST00000567594**	1.5807	0.0398	2.16138	0.00785
**ENST00000483977**	3.04165	0.00405	2.14958	0.00405
**TCONS_00154769**	1.77649	0.0155	2.1135	0.0064
**ENST00000605506**	2.41126	0.00015	2.07759	0.00075
**ENST00000599335**	1.71959	0.0172	2.06368	0.00905
**ENST00000609770**	1.93864	0.0019	1.97419	0.00175
**ENST00000478998**	2.45407	0.0001	1.72076	0.00635
**ENST00000466734**	2.63562	0.0014	1.62634	0.03215
**ENST00000452399**	1.63025	0.03915	1.49795	0.04585
**ENST00000451139**	-1.60294	0.0217	-1.89807	0.0241
**TCONS_00288858**	-2.76911	0.0031	-2.2358	0.0146
**TCONS_00288795**	-2.63417	0.00085	-2.45192	0.00765
**ENST00000519278**	-2.16192	0.0141	-2.61872	0.0084
**ENST00000500447**	-2.30559	0.003	-3.11472	0.00045
**TCONS_00219541**	-1.93845	0.0108	-3.148	0.00555
**ENST00000495810**	-1.95557	0.0113	-4.33066	0.03485

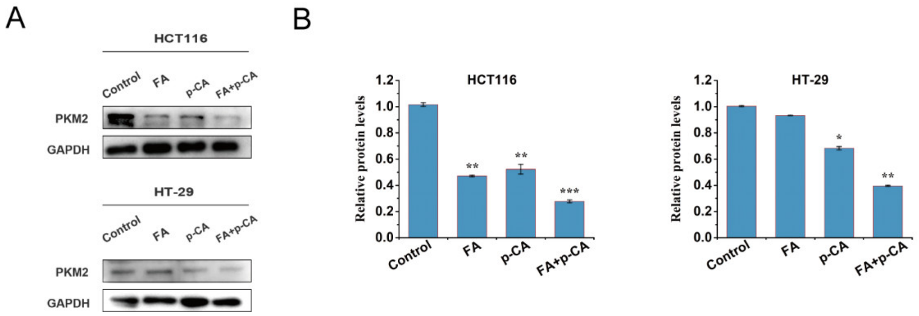

Figure S1. The effects of FA, p-CA and FA + p-CA on PKM2 expression. The HCT116 and HT-29 cells were respectively exposed to 120 μg/mL FA, p-CA and FA + p-CA for 48 h. (**A**) The expression of PKM2 protein was detected by western blot. (**B**) Relative protein levels were analyzed using ImageJ software. Data represented as means ± SEM. *$p < 0.05$, **$p < 0.01$, ***$p < 0.001$.
